# Environmental potassium regulates bacterial flotation, antibiotic production and turgor pressure in *Serratia* through the TrkH transporter

**DOI:** 10.1111/1462-2920.14637

**Published:** 2019-05-13

**Authors:** Alex Quintero‐Yanes, Rita E. Monson, George P. C. Salmond

**Affiliations:** ^1^ Department of Biochemistry University of Cambridge Hopkins Building, Downing Site. Cambridge CB2 1QW UK

## Abstract

*Serratia* sp. strain ATCC 39006 (S39006) can float in aqueous environments due to natural production of gas vesicles (GVs). Expression of genes for GV morphogenesis is stimulated in low oxygen conditions, thereby enabling migration to the air–liquid interface. Quorum sensing (via SmaI and SmaR) and transcriptional and post‐transcriptional regulators, including RbsR and RsmA, respectively, connect the control of cell buoyancy, motility and secondary metabolism. Here, we define a new pleiotropic regulator found in screens of GV mutants. A mutation in the gene *trkH*, encoding a potassium transporter, caused upregulation of GV formation, flotation, and the prodigiosin antibiotic, and downregulation of flagellar motility. Pressure nephelometry revealed that the mutation in *trkH* affected cell turgor pressure. Our results show that osmotic change is an important physiological parameter modulating cell buoyancy and antimicrobial production in S39006, in response to environmental potassium levels.

## Introduction

Bacterial gas vesicles (GVs) are hollow intracellular proteinaceous structures permeable to dissolved gases (Walsby, 1994). Some haloarchea, photosynthetic and heterotrophic bacteria assemble GVs with varying shapes for upward migration and flotation at different depths in aquatic environments (Ramsay *et al.,* 2011; Pfeifer, 2012). These nanostructures are formed mainly by polymers of the small hydrophobic protein, GvpA, and, in most cases, are covered on their outer surface by the hydrophilic protein GvpC to increase resistance to pressure imbalances (Hayes *et al.,* 1988, 1992; Englert and Pfeifer, 1993 *;* Pfeifer, 2012).

At late stages of growth, intracellular GVs accumulate and aggregate in the cytoplasm and the gas space within the GVs causes refraction of light (Walsby, 1994). This refraction leads to a distinctive colony opacity in gas ‘vacuolated’ bacteria, and the phase bright structures (GVs) can be seen in individual cells when observed under phase contrast microscopy (PCM) (Ramsay *et al.,* 2011). These features have enabled facile screening for GV‐defective colonies (which appear translucent) in mutagenesis experiments (Ramsay *et al.,* 2011; Monson *et al.,* 2015; Lee *et al*., 2017). External pressure changes can cause GV collapse and a consequent loss of buoyancy and light refraction in cells. The pressure needed for GV collapse can be measured using nephelometry in cultures subjected to injection of compressed gases (e.g. nitrogen) (Holland and Walsby, 2009; Tashiro *et al.,* 2016). Furthermore, the pressure nephelometry technique applied to hypotonic (turgid) and hypertonic cultures of gas vacuolated bacteria allows a robust assessment of the cell turgor pressure (Walsby, 1994).

**Figure 1:**
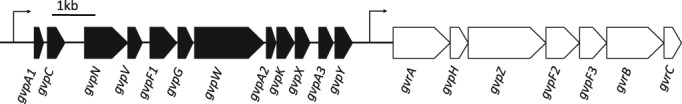
GV genetic cluster. Genes in the *gvpA1*‐*gvpY* and *gvrA*‐*gvrC* operons are represented as thick black and white arrows respectively. Promoters are shown as thin arrows upstream of each operon. This figure is adapted from Ramsay *et al*. (2011).

*Serratia* sp. ATCC 39006 (S39006) is the only enterobacterium, to the best of our knowledge, thus far reported to produce GVs naturally. GV proteins in S39006 are encoded by a cluster composed of 19 genes organized in two contiguous operons (*gvpA1‐gvpY* and *gvrA‐gvrC*) (Fig. 1). These operons are under control of independent promoters upstream of *gvpA1* and *gvrA* and contain genes involved in structural (*gvpA1*, *gvpA2*, *gvpA3*, *gvpC*, *gvpF1*, *gvpF2*, *gvpF3*, *gvpG*, *gvpK*, *gvpN*, *gvpV*), regulatory (gvrA, *gvrB* and *gvrC*) and currently unknown roles (*gvpH*, *gvpW*, *gvpY* and *gvpZ*) (Ramsay *et al.,* 2011; Tashiro *et al.,* 2016). Transcription of the *gvpA1‐gvpY* operon is positively regulated in microaerophilic conditions, suggesting that oxygen depletion is an environmental cue that triggers GV formation for migration to‐, and persistence at, the air–liquid interface. Cognate regulators GvrA, GvrB and GvrC, encoded by the *gvrA‐gvrC* operon, are essential for expression of the *gvpA1‐gvpY* operon (Ramsay *et al.,* 2011; Tashiro *et al.,* 2016). Both deletions and overexpression of *gvrA*, *gvrB* and *gvrC* have negative impacts on *gvpA1* expression (Monson *et al.,* 2016; Tashiro *et al.,* 2016).

Other transcriptional and post‐transcriptional regulators also control GV gene expression (Ramsay *et al.,* 2011; Tashiro *et al.,* 2016; Lee *et al.,* 2017). At low cell densities a LuxR‐family quorum sensing transcription factor (SmaR) inhibits *gvpA1‐gvpY* expression through direct binding to its promoter and, indirectly, through repression of *gvrA‐gvrC* expression. However, when cell population density rises the autoinducer N‐butanoyl‐L‐homoserine lactone (BHL; produced by SmaI) accumulates and binds to SmaR to de‐repress transcription of the GV operons (Ramsay *et al.,* 2011; Tashiro *et al.,* 2016). In addition to quorum sensing, the mRNA‐binding protein, RsmA (the homologue of the *Escherichia coli* CsrA protein) and the ribose operon repressor, RbsR, are also involved in gene regulation for GV production in S39006 (Ramsay *et al.,* 2011; Lee *et al.,* 2017). RsmA and RbsR are positive regulators of the *gvrA‐gvrC* operon and connect cell buoyancy regulation with carbon metabolism.

GV production is co‐regulated in S39006 with flagellar motility and secondary metabolite production via SmaI/SmaR, RsmA and RbsR (Thomson *et al.,* 2000; Slater *et al.,* 2003; Fineran *et al.,* 2005b; Williamson *et al.,* 2008; Ramsay *et al.,* 2011; Wilf *et al.,* 2011; Lee *et al.,* 2017). These regulators control swimming and swarming motility, and antimicrobials such as the β‐lactam antibiotic, 1‐carbapen‐2‐em‐3‐carboxylic acid (a carbapenem) and the red tripyrrole pigment, 2‐methyl‐3‐pentyl‐6‐methoxyprodigiosin (prodigiosin; a prodiginine class molecule with antibacterial, antifungal and antiprotozoal properties) (Coulthurst *et al.,* 2005; Williamson *et al.,* 2006).

Screening for GV mutant colonies in this study led to the identification of a mutant carrying a transposon insertion in the low‐affinity potassium transporter gene, *trkH*. This mutation affected expression of GV biogenesis genes (hence cell buoyancy), turgor pressure, motility and antibiotic production, confirming that potassium availability is an important signal controlling S39006 physiology and behaviour.

## Results

Previous studies on S39006 GV production screened for translucent mutants in the prodigiosin negative strain NWA19 (*Δ* *pigC*) (Ramsay *et al.,* 2011; Lee *et al.,* 2017). We focused this screen on transconjugants that appeared more opaque than NWA19 to try to identify novel negative regulators of GV production. After screening 14 352 colonies, we found a hyper‐opaque mutant (AQY107). The transposon in AQY107 was located in the 3′ region of an ORF sharing high identity and similar genomic context with the low‐affinity potassium transporter gene, *trkH*, from different enterobacteria (Supporting Information Fig. S1).

**Figure 2:**
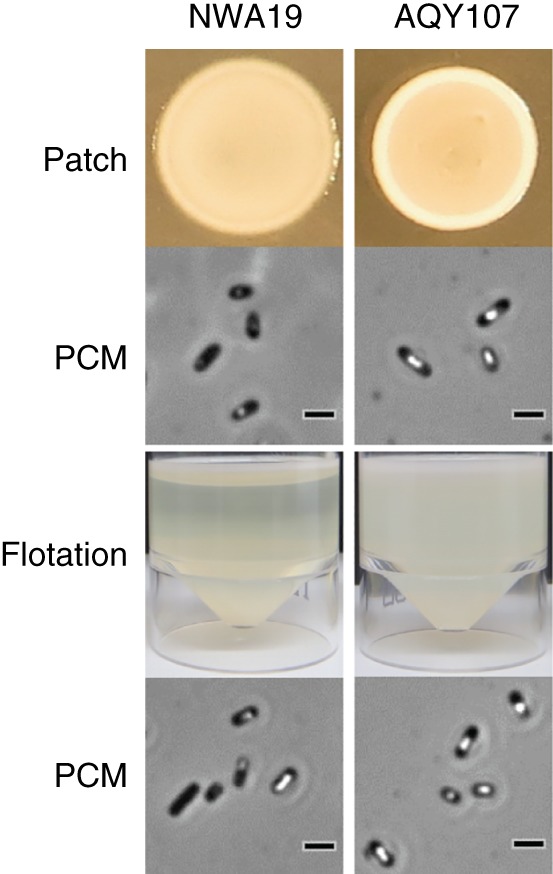
The *trkH* mutation altered patch morphology, flotation and GV formation in S39006. Normalized cultures of NWA19 (*Δ* *pigC*) and AQY107 (*Δ* *pigC trkH*::TnKRCPN1) (Supporting Information Table S1) were spotted on LBA plates to grow cell patches and assess their opacity. PCM images from cells in patches and static cultures (flotation assays) were taken to assess GV formation. All images are representative of biological replicates (*n* = 3). Scale bars in PCM images correspond to 1 μm. [Color figure can be viewed at wileyonlinelibrary.com]

**Figure 3:**
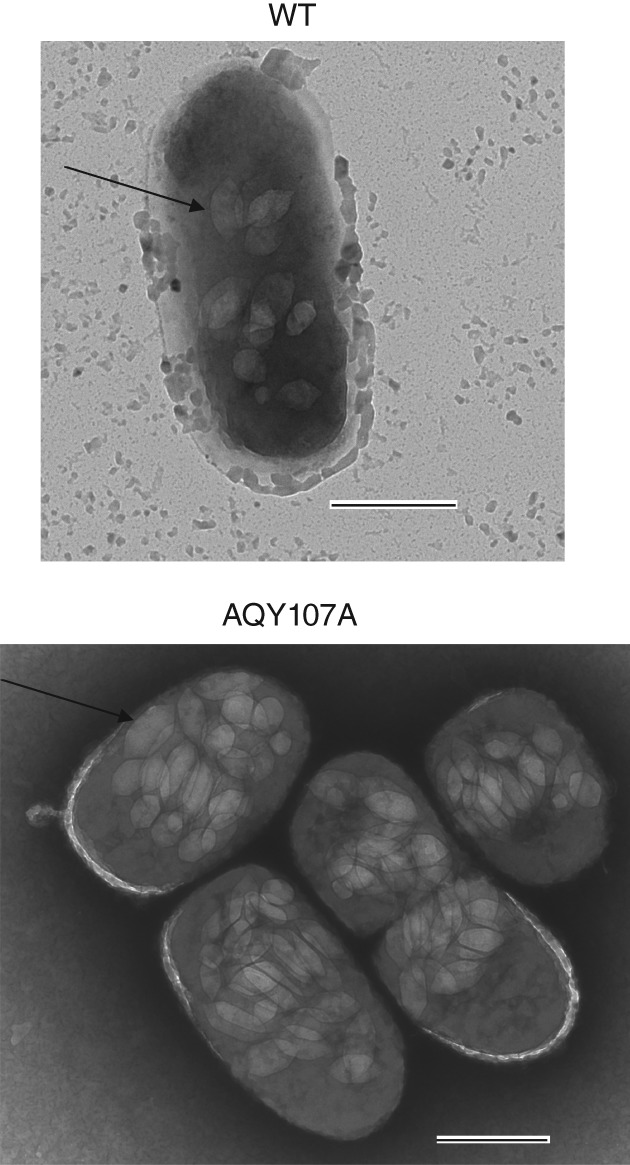
Mutation of *trkH* results in hyper‐production of GVs. TEM images of a WT single cell (top) and a group of AQY107A (*trkH*::TnKRCPN1) (Supporting Information Table S1) cells with GVs. Black arrows indicate GVs. Scale bars correspond to 500 nm.

To confirm our initial observations, we first used φOT8 to transduce the *trkH* mutation back into NWA19 and WT strains and then assessed colony opacity, GV formation and buoyancy in the transductants (Fig. 2). Patches of bacterial cultures with normalized cell number showed that the *trkH* mutant colonies appeared hyper‐opaque; cells in static liquid cultures remained buoyant, and cells from solid and liquid cultures produced more GVs. In contrast, NWA19 cells produced less opaque patches, failed to float and settled to the bottom after 10 days, and produced fewer GVs as seen by PCM. We also analysed GV formation using transmission electron microscopy (TEM). As expected, *trkH* mutants hyper‐produced GVs, whereas moderate production was observed in the WT strain (Fig. 3).

### *Expression of* trkH *down‐regulates gene expression for GV formation*

**Figure 4:**
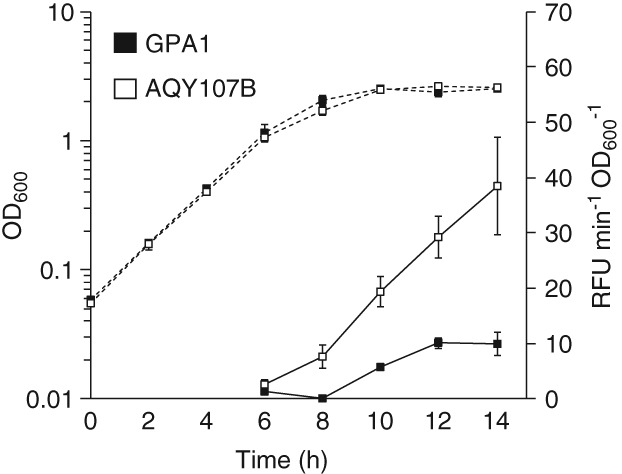
Mutation of *trkH* significantly increases *gvpA1* expression. Growth of GPA1 (*gvpA1*::*uidA*) and AQY107B (*gvpA1*::*uidA*, *trkH*::TnKRCPN1) (Supporting Information Table S1) reporter strains (dotted lines) and β‐glucuronidase reporter activity (continuous lines). Growth was measured as OD_600_ and gene reporter activity as RFU min^−1^ OD_600_^−1^. These data represent the average value of biological replicates (*n* = 3, error bars show standard deviation).

The *gvpA1‐gvpY* operon codes for various proteins important for formation, shape and strengthening of GVs (Ramsay *et al.,* 2011; Tashiro *et al.,* 2016). We measured transcription activity throughout growth in a *gvpA1*::*uidA* reporter fusion strain carrying the transposon insertion in *trkH* (AQY107B) (Supporting Information Table S1). The *trkH* mutation did not have significant impacts on growth in LB media (Fig. 4, Supporting Information Table S2). However, expression of *gvpA1*, measured as enzymatic activity of the β‐glucuronidase reporter, was significantly higher at late‐exponential and stationary phase in the *trkH* background (ANOVA results: F = 86.86 > F_crit_ = 4.35; *p‐*value 1.02 × 10^−8^) (Fig. 4). This result indicated that the TrkH potassium transporter was important for regulation of GV gene expression in WT S39006; with mutation causing hyper‐production of the buoyancy structures.

**Figure 5:**
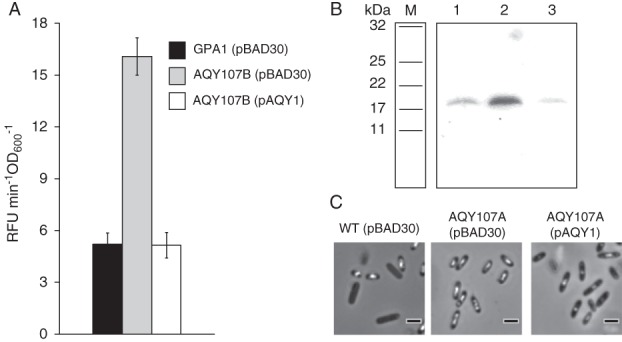
Ectopic expression of *trkH* in mutants has negative impacts on *gvpA1* expression and GV formation. A. Complementation of *gvpA1* expression in the *trkH* mutant. The β‐glucuronidase reporter activity in strains GPA1 (*gvpA1*::*uidA*) and AQY107B (*gvpA1*::*uidA*, *trkH*::TnKRCPN1) (Supporting Information Table S1) containing the empty vector (pBAD30) and AQY107B with pAQY1 (pBAD30 + *trkH*) (Supporting Information Table S1) was measured after 10 h of growth. These data represent the average value of biological replicates (*n* = 3, error bars show standard deviation). B. Western blot with a GvpC antibody in whole cell soluble protein samples. Lane M shows the corresponding size markers (Colour pre‐stained protein standard, 11–225 kDa, NEB), lanes 1, 2 and 3 show the GvpC levels in WT (pBAD30), AQY107A (*trkH*::TnKRCPN1) (pBAD30) and AQY107A (pAQY1) (Supporting Information Table S1) respectively. D. Complementation of GV formation in cells grown overnight in LBA plates. Scale bars correspond to 1 μm. All assays were performed with cells grown in media supplemented with ampicillin and arabinose.

To confirm the impact of *trkH* on GV production, we assessed *gvpA1* expression, GV protein detection and formation in trans‐complemented mutants. Figure 5A shows that ectopic expression of *trkH* under control of an arabinose inducible promoter in AQY107B (Supporting Information Table S1) significantly reduced the β‐glucuronidase activity compared with that in the mutant carrying an empty vector. We also assessed the production of GvpC; a protein encoded by the *gvpA1‐gvpAY* operon and important for strengthening the assembled GVs. As expected, the *trkH* mutant carrying an empty vector showed hyperproduction of GvpC, and this was reduced in the trans‐complemented mutant (Fig. 5B). We corroborated these results by analysing GVs in PCM and observed that complementation of *trkH* resulted in reduction of GV production (Fig. 5C).

The *gvpA1‐gvpY* operon and consequent GV formation are controlled by cognate transcriptional regulators GvrA, GvrB and GvrC expressed from the *gvrA*‐*gvrC* operon (Fig. 1) (Ramsay *et al.,* 2011; Monson *et al.,* 2016; Tashiro *et al.,* 2016). Therefore, we assessed whether the *trkH* mutation may act on GV synthesis via *gvrA‐gvrC* expression. The β‐glucuronidase reporter activity in a *gvrA*::*uidA* fusion strain carrying the transposon insertion in *trkH* (AQY107C) (Supporting Information Table S1) did not show significant alterations (Supporting Information Fig. S2A). This result led us to test whether the mutation in *trkH* might bypass mutations in GV essential genes in the *gvrA*‐*gvrC* operon, such as *gvrA*, *gvpF2*, *gvpF3*, *gvrB* and *gvrC* (Supporting Information Figure S2B). The patch phenotype and PCM of double mutants showed that, although *trkH* did not impact the promoter activity of the *gvrA*‐*gvrC* operon, the essential genes in this operon were required for hyperproduction of GVs in the *trkH* single mutant.

### *Environmental potassium controls GV gene expression and morphogenesis through TrkH*

**Figure 6:**
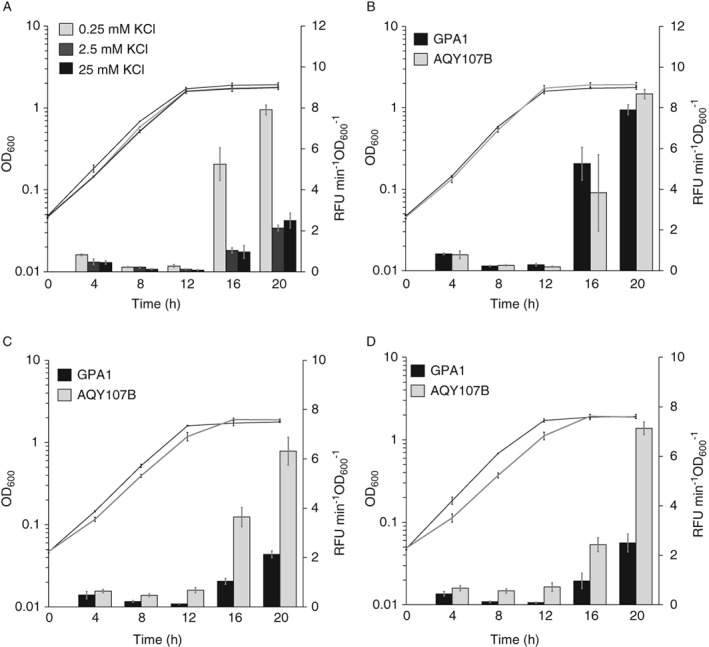
TrkH controls potassium‐dependent expression of *gvpA1*. Growth (lines) was measured as OD_600_ and β‐glucuronidase reporter activity (bars) as RFU min^−1^OD_600_^−1^. A. Growth and reporter activity in GPA1(*gvpA1*::*uidA*) (Supporting Information Table S1) grown in minimal media supplemented with 0.25 mM, 2.5 mM and 25 mM KCl. ANOVA analysis of the β‐glucuronidase reporter activity from 4 to 20 h in cells grown in 0.25 and 2.5 mM KCl F = 73.30 > F_crit_ = 4.08, *p*‐value 1.39 × 10^−10^; in 0.25 and 25 mM KCl F = 69.38 > F_crit_ = 4.08; *p*‐value 2.84 × 10^−10^; and in 2.5 and 25 mM KCl F = 0.59 < F_crit_ = 4.08; *p*‐value 0.45. B–D. Growth and reporter activity in GPA1 (black) and AQY107B (*gvpA1*::*uidA*, *trkH*::TnKRCPN1) (grey) strains (Supporting Information Table S1) in minimal media supplemented with (B) 0.25 mM, (C) 2.5 mM and (D) 25 mM KCl. ANOVA analysis of the β‐glucuronidase reporter activity from 2 to 20 h of growth with (B) 0.25 mM: F = 0.12 < F_crit_ = 4.08; *p*‐value 0.73, (C) 2.5 mM: F = 518.89 > F_crit_ = 4.08; *p*‐value 1.61 × 10^−24^ and (D) 25 mM KCl: F = 521.89 > F_crit_ = 4.08; *p*‐value 1.45 × 10^−24^. The data represent the average and standard deviation (error bars) of three biological replicates.

TrkH is a low‐affinity potassium uptake transporter active at relatively high substrate concentrations, compared with other systems in *E. coli* (Rhoads *et al.,* 1976; Schlösser *et al.,* 1995). We assessed growth, *gvpA1* expression, flotation and GV formation in WT, reporter fusion and *trkH* mutant strains GPA1 (*gvpA1*::*uidA*), AQY107A (*trkH*::TnKRCPN1) and AQY107B (*gvpA1*::*uidA*, *trkH*::TnKRCPN1) (Supporting Information Table S1) grown in minimal media at different potassium concentrations (0.25, 2.5 and 25 mM KCl). First, we noticed that low‐to‐mid potassium concentrations (0.25 and 2.5 mM KCl) had significant negative impacts on GPA1 growth (Fig. 6A, Supporting Information Table S2). GPA1 showed similar negative impacts on β‐glucuronidase reporter activity throughout growth in mid‐to‐high concentrations of KCl (2.5 and 25 mM), whereas in lower (0.25 mM) KCl concentrations, the reporter activity increased significantly during stationary phase (Fig. 6A). Growth and reporter activity in the *trkH* mutant did not vary significantly in 0.25 mM KCl (Fig. 6B, Supporting Information Table S2). In contrast, although growth was affected, the reporter activity increased significantly from mid‐exponential phase in the *trkH* mutant in 2.5 and 25 mM KCl (Fig. 6C and D, Supporting Information Table S2). We confirmed the effect of potassium and the *trkH* mutation on GV gene expression by measuring *gvpA1* expression in minimal media supplemented with potassium phosphate buffer as an alternative K^+^ source to KCl (Supporting Information Fig. S3). Similar to the expression assays in minimal media with KCl, the β‐glucuronidase activity increased in *trkH* mutants grown at high potassium concentrations.

**Figure 7:**
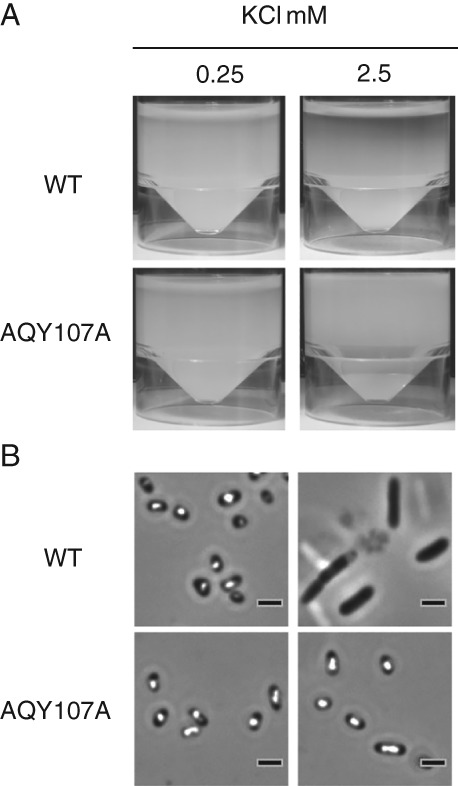
TrkH controls potassium‐dependent regulation of flotation and gas vesicle formation. WT and AQY107A (*trkH*::TnKRCPN1) (Supporting InformationTable S1) cells grown in minimal media with 0.25 mM or 2.5 mM KCl. A. Flotation assay. B. PCM of cells grown in minimal media. Scale bars correspond to 1 μm.

PCM analysis of cells grown in minimal media at different potassium concentrations indicated that GV formation was absent in WT cells at 2.5 mM KCl (Supporting Information Fig. S4). Mutants grown under the same conditions formed GVs in exponential phase (10 h), whereas in low KCl concentrations, these structures were detected at stationary phase in both WT and mutant (Supporting Information Fig. S4). Assays of static liquid cultures in minimal media also confirmed that GV formation and flotation, although absent in the WT at higher potassium concentrations, were pronounced in the mutant (Fig. 7). Interestingly, WT and *trkH* mutant cultures remained buoyant, producing cells with GVs at low potassium concentrations (Fig. 7). Expression of the *gvpA1‐gvpY* operon, GV morphogenesis and flotation decreased at higher potassium concentrations, whereas the opposite effect was observed in the *trkH* mutant. These results showed that potassium, imported via the TrkH transporter, acts as a key environmental signal, regulating cell buoyancy in WT S39006.

### *The* trkH *mutation still has physiological impacts in low‐aeration conditions*

Upward migration and flotation in aqueous environments is facilitated by biogenesis of GVs. This is an important adaptive strategy in various halobacteria, heterotrophs and aerobes that enables migration to maximize access to oxygen (Walsby, 1994; Ramsay *et al.,* 2011). As seen in *trkH*‐dependent regulation of GVs, microaerophilic conditions up‐regulate the transcription of the *gvpA1*‐*gvpY* operon, but not the *gvrA*‐*gvrC* operon, in S39006 (Ramsay *et al.,* 2011). Consequently, we determined whether the overexpression of the *gvpA1*‐*gvpY* operon observed in the *trkH* mutants was also manifested under oxygen‐depleted conditions. The β‐glucuronidase reporter activity in cultures grown under low aeration was higher in AQY107B than in GPA1 (Supporting Information Fig. S5). This suggested that negative regulation of GV and flotation due to environmental potassium (operating through the TrkH transporter) is active in oxygen‐limited environments.

### *The mutation in* trkH *is pleiotropic*

Cell buoyancy control is physiologically connected with motility and antimicrobial production through global pleiotropic regulators in S39006 (Ramsay *et al.,* 2011; Lee *et al.,* 2017). We noticed that AQY107A showed moderately impaired flagellar motility when compared with WT, while ectopic expression of wild‐type *trkH* restored swimming motility in the mutant (Supporting Information Fig. S6A). This result suggested that TrkH enables potassium‐dependent positive regulation of motility in S39006.

Extraction of prodigiosin from cells at stationary phase of growth showed that pigment production in the *trkH* background was higher than in the WT strain, and genetic complementation of AQY107A reduced production (Supporting Information Fig. S6B). Pigment levels were also reduced at higher potassium concentrations in WT samples and elevated in the *trkH* mutant (Supporting Information Fig. S7A). Furthermore, *β*‐galactosidase reporter activity in a *pigA*::*lacZ* strain confirmed that extracellular potassium affects prodigiosin biosynthesis gene expression at the transcriptional level (Supporting Information Fig. S7B). These results confirmed that potassium uptake through the TrkH transporter also modulates prodigiosin biosynthesis.

### *The mutation in* trkH *affects cell turgor pressure*

**Figure 8:**
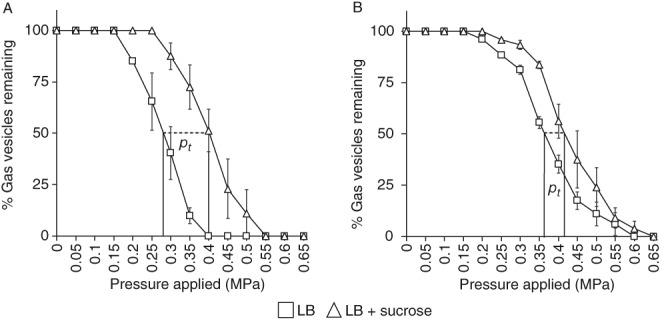
The *trkH* mutation affects turgor pressure. Pressure nephelometry of (A) WT, (B) AQY107A (*trkH*::TnKRCPN1) (Supporting Information Table S1) cultures was performed in turgid (LB) and hypertonic (LB + 0.35 M sucrose) conditions. Turgor pressure (pt) values are indicated for each strain in the text. These data represent the average vale and standard deviation (error bars, ±) of biological replicates (*n* = 3).

Pressure nephelometry experiments with the cyanobacterium *Anabaena flos‐aquae* showed that potassium uptake increased turgor pressure and, hence, caused GV collapse (Allison and Walsby, 1981). Given that TrkH facilitates potassium uptake in S39006, we expected cell turgor to be reduced in the *trkH* mutant. Therefore, we assessed the collapse of GVs in LB (turgid medium) and LB with sucrose (hypertonic medium) in the mutant. The difference between the mean critical collapse pressure (when 50% of the GVs collapse) in turgid and hypertonic cultures of WT and *trkH* cells showed that turgor pressure in WT samples was similar to previous measurements (*p*_*t*_ = 0.121 MPa, ± 0.012) (Tashiro *et al.,* 2016) but significantly reduced in the *trkH* mutant (*p*_*t*_ = 0.061 MPa, ± 0.03) (Fig. 8A and B).

## Discussion

Multiple environmental cues, such as carbon source, amino acids, nitrogen, phosphate and light, are known to affect flotation in different bacteria to allow adaptive positioning and vertical migration in water columns (Walsby and Klemer, 1974; Konopka, 1977; Oliver and Walsby, 1984; Brookes and Ganf, 2001). In *A. flos‐aquae*, light‐dependent regulation of cell buoyancy involves rapid potassium uptake (Allison and Walsby, 1981). Exposure to light, through unknown mechanisms, increases intracellular K^+^ and, consequently, turgor pressure rises sufficiently to induce GV collapse. Unlike *A. flos‐aquae*, S39006 is a non‐photosynthetic heterotroph, but experiments in this study have shown that the potassium transporter TrkH also controls turgor pressure and biosynthesis of GVs in this bacterium. It is possible that TrkH controls osmotic transitions via rapid potassium uptake in S39006 to facilitate downward migration in the water column, via genetic repression of GVs.

In aerobic bacteria, GV biosynthesis enables adaptive migration into oxygenated niches, such as air–liquid interfaces (Walsby, 1976; Ramsay *et al.,* 2011). Therefore, we considered the possibility that potassium‐mediated repression of GV production may not be active or necessary in oxygen‐depleted conditions. However, transcription assays indicated that TrkH‐dependent regulation was active in both aerated and microaerophilic environments (Figs. 4 and Supporting Information Fig. S5) implying that potassium availability may be a more important environmental cue than oxygen.

Also, potassium and oxygen have been reported previously as important chemo‐attractants stimulating flagellar motility in free‐living cells (Armitage, 1997; Humphries *et al.,* 2017). Here, we showed that TrkH is a positive regulator of swimming motility in S39006 under conditions when GVs are downregulated (Supporting Information Fig. S6). TrkH might be important in modulating potassium‐dependent behavioural transitions in S39006, between passive buoyant and active motile states. High extracellular potassium concentrations cause membrane depolarization leading to enhanced potassium influx. The resultant osmotic shift causes cell hyperpolarization and the increased proton motive force (PMF) powers flagellar rotation (Humphries *et al.,* 2017). Potassium‐rich environments may therefore favour swimming over flotation, due to their capacity to generate PMF and downregulate GVs. In addition, potassium flux might have significant impacts on how S39006 populations are distributed in aquatic environments because swimming cells can move in different directions to explore new environments, whereas GV‐producing bacteria are limited to vertical movement (Walsby, 1994).

Similar to GVs, the antimicrobial prodigiosin was downregulated in high potassium concentrations (Supporting Information Figs. S6 and S7). Interestingly, high phosphate concentrations and different carbon sources such as ribose and gluconate also have negative impacts on prodigiosin production in S39006 (Fineran *et al.,* 2005a; Lee *et al.,* 2017). Previous work in *Serratia marcescens* also showed that carbon sources, such as glucose and maltose, have negative impacts on prodigiosin expression (Haddix *et al.,* 2008). Moreover, prodigiosin production in *S. marcescens* is inversely correlated with growth rate and ATP synthesis and so it has been suggested that prodigiosin may have a physiological role as an ‘energy spilling’ molecule involved in reduction of ATP levels in cells (Haddix *et al.,* 2008). As potassium is essential for ATPase functioning through PMF (Maloney *et al.,* 1974), the results in this study are consistent with this proposed physiological role for prodigiosin (Haddix *et al.,* 2008).

A recent study reported that potassium uptake via the Trk system stimulated virulence in the phytopathogen, *Pectobacterium wasabiae*, via control of *rsmB* expression (Valente and Xavier, 2016). The small sRNA *rsmB* binds to RsmA to antagonize the target mRNA binding activity of the latter in a widespread post‐transcriptional regulation system (Romeo and Babitzke, 2018). However, in contrast to the study with *P. wasabiae*, we found that in S39006 mutation of *trkH* does not affect either *rsmB or rsmA* expression (Supporting Information Fig. S8). These results indicate that the TrkH‐dependent signal transduction pathway in S39006 must be different from that in *P. wasabiae*.

In *E. coli*, multiple transporters, such as TrkH/G, Kup (TrkD) and KdpFABC, are involved in potassium transport at different environmental concentrations (Rhoads *et al.,* 1976; Bossemeyer *et al*., 1989). S39006 contains one single‐gene copy of the transporter of the Trk system (*trkH*). Orthologues of TrkD and KdpFABC, which are higher affinity potassium transport systems compared with TrkH, are also present in S39006. Thus, as seen in *E. coli*, it is likely that the low‐affinity Trk system controls potassium transport at high environmental concentrations, whereas TrkD and KdpFABC may be acting in potassium‐depleted conditions. Interestingly, it has been shown that high potassium concentrations repress the expression of the high‐affinity potassium transport system KdpFABC in *E. coli* (Rhoads *et al.,* 1976). High extracellular potassium concentrations also inhibit the two‐component system, KdpDE, which is known to control transcription of the *kdpFABC* operon (Laermann *et al.,* 2013). Orthologues of this two‐component system are also present in S39006. Recently, the Kdp and Trk systems have been linked physiologically. Work on a TrkH homologue, TrkJ, from *Azorhizobium caulinodans* showed that this transporter facilitated potassium‐dependent repression of *kdpFABC* expression through an unknown mechanism (Siarot *et al.,* 2017). Interestingly, experiments in Gram‐positive bacteria showed that the sensor kinase, KdpD is inhibited after cyclic‐di‐AMP (c‐di‐AMP) binding (Bai *et al.,* 2014; Moscoso *et al.,* 2016). Multiple recent reports lately have shown the impact of c‐di‐AMP in osmoregulation and its interaction with a TrkH effector protein (Zarrella *et al.,* 2018; Pham and Turner, 2019; Quintana *et al.,* 2019). However, c‐di‐AMP is not synthesized in Gammaproteobacteria (Commichau *et al.,* 2018), such as S39006. Considering the absence of diadenylate cyclase homologues and the fact that *trkH* does not regulate *rsmB* expression in S39006, further studies will be required to uncover the regulatory interactions between TrkH and KdpDE, and potassium‐dependent regulation of cell buoyancy, motility and secondary metabolism in this bacterium.

Environmental potassium availability is an important factor affecting multiple aspects of bacterial physiology and ecology (Tokuda *et al.,* 1981; Epstein, 2003; Podell *et al.,* 2014; Prindle *et al.,* 2015; Gundlach *et al.,* 2017; Humphries *et al.,* 2017). There have been reports describing how potassium flux through the Trk system influences diverse processes such as protein secretion, virulence and resistance to antimicrobial peptides and aminoglycoside antibiotics in bacterial pathogens of plants and man (Groisman *et al.,* 1992; Laasik *et al.,* 2005; Su *et al.,* 2009; Valente and Xavier, 2016). This study of the TrkH system in S39006 has shown that the transporter is also a significant modulator of cell turgor, organelle morphogenesis, buoyancy, motility and bioactive secondary metabolite biosynthesis. Moreover, it confirmed that extracellular potassium can act as an important environmental cue repressing specific gene expression to modulate bacterial physiology and adaptive behaviour. Future experiments will dissect the signal transduction pathway from titration of environmental potassium levels to secondary metabolite production, organelle biogenesis and the resulting bacterial cell population phenomenon of flotation.

## Experimental procedures

### *Bacterial strains, media and growth conditions*

S39006 strains (Supporting Information Table S1) were grown at 30°C in lysogeny broth (LB) (10 g l^−1^ tryptone, 5 g l^−1^ yeast extract, 5 g l^−1^ NaCl) in liquid or minimal media (0.1% NH_4_SO_4_, 0.41 mM MgSO_4_, 0.2% glucose, 5.34 g l^−1^ Na_2_HPO_4_, 3.34 g l^−1^ NaH_2_PO_4_) supplemented with either KCl (0.25, 2.5 and 25 mM) or potassium buffer (7 g l^−1^ K_2_HPO_4_, 3.34 g l^−1^ KH_2_PO_4_, pH 7.0) to 0.14, 1.4, 14 mM K^+^ final concentrations. LB with 1.5% (w/v) agar (LBA) was used for growth on solid media. Initially, all seed cultures were collected from single colonies on LBA plates and grown overnight in 5 ml LB in sealed universal tubes on a roller wheel. Thereafter, cultures for assays in minimal media were pelleted and washed twice in cold sterile dH_2_O and diluted to 0.05 OD_600_ in 25 ml minimal media with 0.25 mM KCl for 20 h adaptation. Complementation experiments were performed in LB and LBA with 0.2% arabinose induction for ectopic expression and 50 *μ*g ml^−1^ ampicillin (Ap) selection. Cultures for gene expression and prodigiosin assays were diluted to 0.05 OD_600_ in 250 ml flasks containing 25 ml of LB or minimal media with different potassium concentrations, incubated at 30°C, and grown under aeration by shaking at 215 rpm. Cultures for gene expression assays in microaerophilic conditions were covered with 25 ml sterile mineral oil (Sigma) and shaken at 80 rpm (Ramsay *et al.,* 2011). Cultures for GvpC immunodetection were grown in 500 ml flasks with 50 ml LB. Seed cells for flotation assays were diluted to 0.05 OD_600_, into universal tubes containing 5 ml of LB or minimal media and grown on tube rollers for 24 h. Then, tubes were set upright as static cultures for 10 days on the bench at room temperature.

### *Transposon mutagenesis and screen for GV mutants*

Random transposon mutagenesis with TnKRCPN1 was performed via conjugation with strains NWA19 and *E. coli* β2163 (pKRCPN1) as indicated previously (Monson *et al.,* 2015). Transconjugants were assessed visually for colony opacity. The transposon insertion sites in mutants were identified using random primed PCR (Jacobs *et al.,* 2003; Fineran *et al.,* 2005a) and Sanger DNA sequencing (GATC Biotech) of PCR products with oMAMV2 (Supporting Information Table S1).

### *Bioinformatic analysis*

The transposon insertion site was identified using Artemis 16.0 (Carver *et al.,* 2011) for nucleotide alignment with the genome sequence of S39006 (Fineran *et al.,* 2013). The EMBOS needle protein alignment tool (Li *et al.,* 2015) helped to determine the identity and similarity of the predicted amino acid sequence of the open reading frame (ORF) affected by TnKRCPN1. The gene sequence viewer from NCBI was used to identify upstream and downstream ORFs of *trkH* in different enterobacteria for comparison with S39006.

### *Phage transduction*

The transposon insertion in *trkH* was moved by phage φOT8 transduction into different S39006 strains, as described previously (Evans *et al.,* 2010). Transductants were selected on LB plates with either 25 *μ*g ml^−1^ kanamycin (Km) or 35 *μ*g ml^−1^ chloramphenicol (Cm).

### *Microscopy*

Samples for PCM imaging were prepared as described previously (Ramsay *et al.,* 2011). PCM Images were taken using an Olympus BX‐5 microscope with a 100× oil‐immersion lens and a QICAM monochrome camera adapted to the QCapture Pro‐6 software. Images were processed using ImageJ (Abràmoff *et al.,* 2004). Samples for TEM were prepared for imaging using a carbon‐coated glow‐discharged grid treated with 0.01% poly‐L‐lysine for 2 min, and then 5 μl of undiluted cell suspensions taken from 2 days of static growth in LB were attached for 10 min and rinsed with dH2O. Cells were stained with 2% phosphotungstic acid (pH 7.0) neutralized with KOH. Cell images were obtained using a FEI Tecnai G2 TEM in the Cambridge Advance Imaging Centre, University of Cambridge (Lee *et al.,* 2017).

### *β‐glucuronidase (UidA) and β‐galactosidase (LacZ) assays*

Gene expression in *uidA* and *lacZ* fusion strains was assessed as previously reported (Ramsay *et al.,* 2011; Monson *et al.,* 2015). Enzymatic activity was quantified using a Gemini XPS plate reader following the parameters described previously (Ramsay *et al.,* 2011). The transcription activity at each time point was normalized to culture OD_600_.

### *Plasmid construction*

For construction of pAQY1, *trkH* was amplified with oligos oAQ44 and oAQ45 (Supporting Information Table S1). The resulting PCR product and pBAD30 vector were digested with SacI‐HF and XbaI (NEB) for 3 h at 37°C. Digestion was heat‐inactivated at 65°C for 20 min. Insert and vector were ligated with T4 DNA ligase (Thermo Fisher Scientific) following manufacturer's instructions. Cloning was confirmed by Sanger DNA sequencing (GATC Biotech).

### *GvpC Western blot*

Cells were grown in LB as indicated above for aerated conditions, collected at 16 h, normalized to 2.0 OD_600_, pelleted at 8000 *g* and 4°C, and resuspended in 1.25 ml of CHAPS lysis buffer containing 1X Calbiochem protease inhibitor cocktail set I (Merck) (Coulthurst *et al.,* 2006). The lysis solution was kept on ice and sonicated for 3 cycles × 20 s. Cell debris and insoluble material was pelleted at 13 000 *g* and 4°C. Protein samples were separated using 15% acrylamide SDS gels. Proteins were transferred to an Immobilon‐P PVDF membrane (Merck), washed three times for 5 min with 0.1% (v/v) Tween 20 in phosphate‐buffered saline (PBS), and blotted in 5% (w/v) milk in Tween 20‐PBS (blocking solution) with rabbit GvpC antibody (1:30 000 antibody to blocking solution volume ratio) for 1 h and goat IgG (1:30 000 IgG to blocking solution volume ratio) for 40 min. The GvpC antibody was raised against the MAQLKNIDDSHES peptide, immunized in rabbits (BioGenes GmbH) and was pre‐absorbed to whole protein precipitates from a Δ*gvpC* strain of S39006 before usage (Tashiro *et al.,* 2016).

### *Phenotypic assays*

Bacterial growth in patches was assessed using 10 *μ*l spots of normalized (1.0 OD_600_) cell cultures on LBA plates. Spotted cultures were allowed to dry then plates were incubated overnight. Swimming, prodigiosin and carbapenem assays were done as previously indicated (Slater *et al.,* 2003; Williamson *et al.,* 2008). Swimming complementation assays were done on 120 mm × 120 mm × 17 mm Greiner square dishes (Merck).

### *Pressure nephelometry and turgor pressure measurement*

S39006 strains were grown overnight in sealed universal tubes with 5 ml LB and then set as static cultures for 24 h. Thereafter, GV collapse measurements were performed using a pressure nephelometer (using the same apparatus designed by Holland and Walsby (2009)). Changes in turbidity (nephelometry) caused by GV collapse were taken after gradual pressure injections of 0.05 MPa using compressed N_2_. A blank of 4 ml of media without cells was used to set the millivoltmeter to zero. Afterwards, 0.5 ml of cells from cultures described above were added to the tubes containing either LB (turgid condition) or LB with 0.35 M sucrose (hypertonic condition). The tubes were hermetically sealed and the millivoltmeter set to 100. The proportion of GVs remaining after pressure injections and turgor pressure values were determined as described previously (Tashiro *et al.,* 2016).

## Conflict of interest

The authors do not have any conflict of interest in relation to the work described.

## Supporting information


**Table S1.** Bacterial strains, phage, plasmids and oligonucleotides.
**Table S2.** ANOVA (two‐factor with replication) analysis from growth experiments in Figs 4 and 6.
**Fig. S1.** Bioinformatic analysis of the transposon insertion site in AQY107. Genomic context of TnKRCPN1 insertion site and comparison of TrkH homologous in different enterobacteria. The black arrow indicates the insertion site of the transposon in AQY107 (Δ*pigC*, *trkH*::TnKRCPN1) (Table S1). The disrupted gene in AQY107 and its homologous are highlighted in white. The percentage of identity/similarity of the TrkH proteins is indicated above each homologue.
**Fig. S2.** Effect of *trkH* mutation on the *gvrA*‐*gvrC* operon. A. *gvrA* transcription activity in GRA (*gvrA*::*uidA*) and AQY107C (*gvrA*::*uidA*, *trkH*::TnKRCPN1) (Table S1). Growth (dotted lines) was measured as OD_600_ and gene reporter activity (continuous lines) as RFU min^−1^ OD_600_^−1^. ANOVA analysis of the β‐glucuronidase reporter activity from 6 to 14 h of growth F = 3.17 > F_crit_ = 4.35; p‐value 0.09. These data represent the average value of biological replicates (n = 3, error bars show standard deviation). B. Patch morphology and PCM of patches cells with mutations in *trkH* and GV essential genes from the *gvrA*‐*gvrC* operon. *trkH* mutant cells AQY107D, G, H, E and F in‐frame mutations in *gvrA*, *gvpF2*, *gvpF3*, *gvrB* and *gvrC*, respectively (Table S1). Scale bars correspond to 1 μm.
**Fig. S3.** *gvpA1* expression in minimal media with an alternate potassium source to KCl. Reporter fusion strains GPA1 (*gvpa1*::*uidA*) and AQY107B (*gvpa1*::*uidA trkH*::TnKRCPN1) (Table S1) were grown in minimal media at final concentrations of (A) 0.14 mM, (B) 1.4 mM and (C) 14 mM K^+^ using minimal medium with potassium buffer instead of KCl as a source of K^+^. ANOVA analysis of the β‐glucuronidase reporter activity from 12 to 16 h of growth with (A) F = 4.08 < F_crit_ = 4.74; p‐value 0.066; (B) F = 70.87 > F_crit_ = 4.74; p‐value 2.22*10^–6^, and (C) F = 42.57 > F_crit_ = 4.74; p‐value 2.83*10^–5^. These data represent the average value of biological replicates (n = 3, error bars show standard deviation).
**Fig. S4.** Effect of potassium on gas vesicle formation in WT and *trkH* strains. (A) Growth and (B) gas vesicle formation throughout time in WT and AQY107A (*trkH*::TnKRCPN1) (Table S1) cells grown in the presence of 0.25 mM and 2.5 mM KCl. Images of cells with GVs are framed with black lines. PCM images were taken immediately after OD_600_ measurements. These data represent the average value of biological replicates (n = 3, error bars show standard deviation).
**Fig. S5.** *gvpA1* expression in the *trkH* mutant under microaerophilic conditions. Growth (dotted lines) was measured as OD_600_ and reporter activity (continuous lines) as RFU min^−1^ OD_600_^−1^ in reporter fusion strains GPA1 (*gvpa1*::*uidA*) and AQY107B (*gvpa1*::*uidA trkH*::TnKRCPN1) (Table S1). ANOVA analysis of the β‐glucuronidase reporter activity from 6 to 20 h of growth F = 86.86 > F_crit_ = 4.35; p‐value 1.02*10^–8^. These data represent the average value of biological replicates (n = 3, error bars show standard deviation).
**Fig. S6.** TrkH is a pleiotropic regulator. Complementation of (A) swimming motility and (B) prodigiosin production (A_534_ ml^−1^ OD_600_^−1^) in the *trkH* mutant. WT and AQY107A (*trkH*::TnKRCPN1) carrying pBAD30 (empty vector) (Table S1) were used as controls. AQY107A was complemented with pAQY1 (Table S1). (A) The image is representative of three biological replicates. (B) These data represent the average value of biological replicates (n = 3, error bars show standard deviation).
**Fig. S7.** Potassium and TrkH are negative regulators of prodigiosin production. A. Pigment production of WT and AQY107A (*trkH*::TnKRCPN1) (Table S1). B. β‐galactosidase reporter activity in fusion strain MC2PL (*pigA*::*lacZ*) (Table S1). WT, AQY107A and MCP2L cells were grown in minimal media with different potassium concentrations and measurements were taken after 12 h. These data represent the average value of biological replicates (n = 3, error bars show standard deviation).
**Fig. S8.** The mutation in *trkH* did not alter *rsmB* and *rsmA* transcription. Growth (dotted lines) and the β‐glucuronidase gene reporter acctivity (bars) in (A) *rsmB*::*uidA* and AQY107I (*rsmB*::*uidA*, *trkH*::TnKRCPN1), and (B) NWA64 (*rsmA*::*uidA*) and AQY107J (*rsmA*::*uidA*, *trkH*::TnKRCPN1) (Table S1). Cells were grown in LB media. These data represent the average value of biological replicates (n = 3, error bars show standard deviation).
