# Supplementary material for: Environmental potassium regulates bacterial flotation, antibiotic production and turgor pressure in Serratia through the TrkH transporter
Source: Environ Microbiol. 2019 May 13;21(7):2499–510. doi: 10.1111/1462-2920.14637 (PMC6617781; doi:10.1111/1462-2920.14637)
Supplement: Supplementary file 1 — Table S1. Bacterial strains, phage, plasmids and oligonucleotides. Table S2. ANOVA (two‐factor with replication) analysis from growth experiments in Figs 4 and 6. Fig. S1. Bioinformatic analysis of the transposon insertion site in AQY107. Genomic context of TnKRCPN1 insertion site and comparison of TrkH homologous in different enterobacteria. The black arrow indicates the insertion site of the transposon in AQY107 (ΔpigC, trkH::TnKRCPN1) (Table S1). The disrupted gene in AQY107 and its homologous are highlighted in white. The percentage of identity/similarity of the TrkH proteins is indicated above each homologue. Fig. S2. Effect of trkH mutation on the gvrA‐gvrC operon. A. gvrA transcription activity in GRA (gvrA::uidA) and AQY107C (gvrA::uidA, trkH::TnKRCPN1) (Table S1). Growth (dotted lines) was measured as OD600 and gene reporter activity (continuous lines) as RFU min−1 OD600 −1. ANOVA analysis of the β‐glucuronidase reporter activity from 6 to 14 h of growth F = 3.17 > Fcrit = 4.35; p‐value 0.09. These data represent the average value of biological replicates (n = 3, error bars show standard deviation). B. Patch morphology and PCM of patches cells with mutations in trkH and GV essential genes from the gvrA‐gvrC operon. trkH mutant cells AQY107D, G, H, E and F in‐frame mutations in gvrA, gvpF2, gvpF3, gvrB and gvrC, respectively (Table S1). Scale bars correspond to 1 μm. Fig. S3. gvpA1 expression in minimal media with an alternate potassium source to KCl. Reporter fusion strains GPA1 (gvpa1::uidA) and AQY107B (gvpa1::uidA trkH::TnKRCPN1) (Table S1) were grown in minimal media at final concentrations of (A) 0.14 mM, (B) 1.4 mM and (C) 14 mM K+ using minimal medium with potassium buffer instead of KCl as a source of K+. ANOVA analysis of the β‐glucuronidase reporter activity from 12 to 16 h of growth with (A) F = 4.08 < Fcrit = 4.74; p‐value 0.066; (B) F = 70.87 > Fcrit = 4.74; p‐value 2.22*10–6, and (C) F = 42.57 > Fcrit = 4.74; p‐value 2.83*10–5. These data repr [file EMI-21-2499-s001.pdf]

Environmental potassium regulates bacterial flotation, antibiotic production and turgor pressure in *Serratia* through the TrkH transporter

Authors

Alex Quintero-Yanes, Rita Monson & George Salmond

Supplementary figures, tables and references.

Table S1. Bacterial strains, phage, plasmids and oligonucleotides.

Name	Genetic Information	Reference*
	**Bacteria**	
***Serratia* sp. ATCC 39006 (S39006)**		
WT	Lac$^-$ (LacA), laboratory strain referred to as wild type	1
NWA19	LacA, Δ*pigC*	2
GPA1	LacA, *gvpA1*::TnDS1028-*uidA*, Cm^R	2
GRA	LacA, *gvrA*::TnDS1028-*uidA,* Cm^R	2
Δ*gvrA*	In-frame *gvrA* mutant derivative of WT	3
Δ*gvrB*	In-frame *gvrB* mutant derivative of WT	3
Δ*gvrC*	In-frame *gvrC* mutant derivative of WT	3
Δ*gvpF2*	In-frame *gvpF2* mutant derivative of WT	3
Δ*gvpF3*	In-frame *gvpF3* mutant derivative of WT	3
MCP2L	LacA, *pigA*:: miniTn*5lacZ1,* Km^R	4
NW64	LacA, *rsmA*::TnDS1028-*uidA,* Cm^R	5
rsmB::*uidA*	LacA, *rsmB*::TnDS1028-*uidA,* Cm^R	Lab stock
AQY107	LacA, Δ*pigC, trkH:*:TnKRCPN1, Km^R	This study
AQY107A	LacA*, trkH::*TnKRCPN1, Km^R	This study
AQY107B	LacA, *gvpA1*::TnDS1028-*uidA*, *trkH:*:TnKRCPN1, Cm^R, Km^R	This study
AQY107C	LacA, *gvrA*::TnDS1028-*uidA, trkH:*:TnKRCPN1, Cm^R, Km^R	This study
AQY107D	In-frame *gvrA* mutant derivative of AQY107A	This study
AQY107E	In-frame *gvrB* mutant derivative of AQY107A	This study
AQY107F	In-frame *gvrC* mutant derivative of AQY107A	This study
AQY107G	In-frame *gvpF2* mutant derivative of AQY107A	This study
AQY107H	In-frame *gvpF3* mutant derivative of AQY107A	This study
AQY107I	LacA, *rsmB*::TnDS1028-*uidA, trkH:*:TnKRCPN1, Cm^R, Km^R	This study
AQY107J	LacA, *rsmA*::TnDS1028-*uidA, trkH:*:TnKRCPN1, Cm^R, Km^R	This study

Table S1 continues

Table S1 continued

Name	Genetic Information	Reference*
Escherichia coli		
β2163	(F^-) RP4-2-Tc::Mu Δ*dapA::(erm-pir)*, Em^R	6
Dh5α	F− Ø80*lacZ*ΔM15 Δ(*lacZYA*−argF) U169 *recA1 endA1 hsdR17* (rK−, mK+) *phoA sup*E44 λ− *thi*-1	Life technology
	**Phage**	
φOT8	Generalized ransducing phage for S39006 that recognizes flagella proteins	7
	**Plasmids**	
pKRCPN1	pKRCPN1 plasposon Km^R, Tc^R	8
pBAD30	Expression vector with araBAD promoter, ApR	9
pAQY1	pBAD30 carrying *trkH* from WT	This study
	**Oligonucleotides**	
oMAMV1	GGAATTGATCCGGTGGATG- Transposon specific primer.	10
oMAMV2	GCATAAAGCTTGCTCAATCAATCAC-Transposon specific primer	10
PF106	GACCACACGTCGACTAGTGCNNNNNNNNNNAGAG – Random primed PCR primer 1	10
PF107	GACCACACGTCGACTAGTGCNNNNNNNNNNACGCC- – Random primed PCR primer 2	10
PF108	GACCACACGTCGACTAGTGCNNNNNNNNNNGATAC-– Random primed PCR primer 3	10
PF109	GACCACACGTCGACTAGTGC- – Random primed PCR primer 4	10
oAQ44	GATGAGCTCAAGGAAGGCATCTGTAATGCAC-*trkH* F with SacI restriction sequence	This study
oAQ45	CTATCTAGATTATTCCCGCCAAAAAG-*trkH* R with XbaI restriction sequence	This study

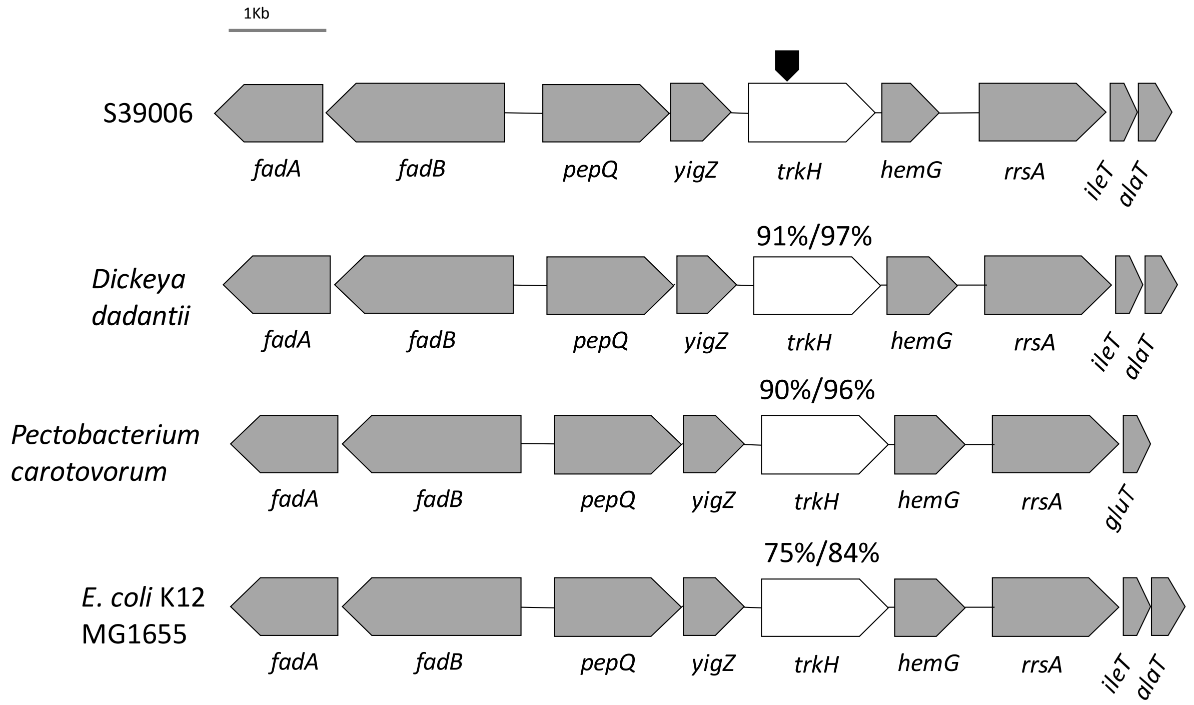

Figure S1. Bioinformatic analysis of the transposon insertion site in AQY107. Genomic context of TnKRCPN1 insertion site and comparison of TrkH homologous in different enterobacteria. The black arrow indicates the insertion site of the transposon in AQY107 (Δ*pigC, trkH:*:TnKRCPN1) (Table S1). The disrupted gene in AQY107 and its homologous are highlighted in white. The percentage of identity/similarity of the TrkH proteins is indicated above each homologue.

Table S2. ANOVA (two-factor with replication) analysis from growth experiments in Figures 4 and 6.

Strain – growth media	Strain- growth media	*F*	*p-value*
GPA1 - LB	AQY107B - LB	0.92	0.34
GPA1 - 0.25	GPA1- 2.5	0.094	0.76
GPA1 - 0.25	GPA1 - 25	53.79	$6.45x10^{-9}$
GPA1 - 2.5	GPA1 – 25	48.92	$1.91x10^{-8}$
GPA1 - 0.25	AQY107B – 0.25	2.65	0.11
GPA1 - 2.5	AQY107B – 2.5	57.40	$2.99x10^{-9}$
GPA1 - 25	AQY107B - 25	182.85	$1.66\ x10^{-16}$

Numeric values for growth media correspond to minimal media with either 0.25, 2.5 or 25 mM KCl. F_{crit} = 4.20 for ANOVA analysis in growth assays in LB, and F_{crit} = 4.08 in minimal media.

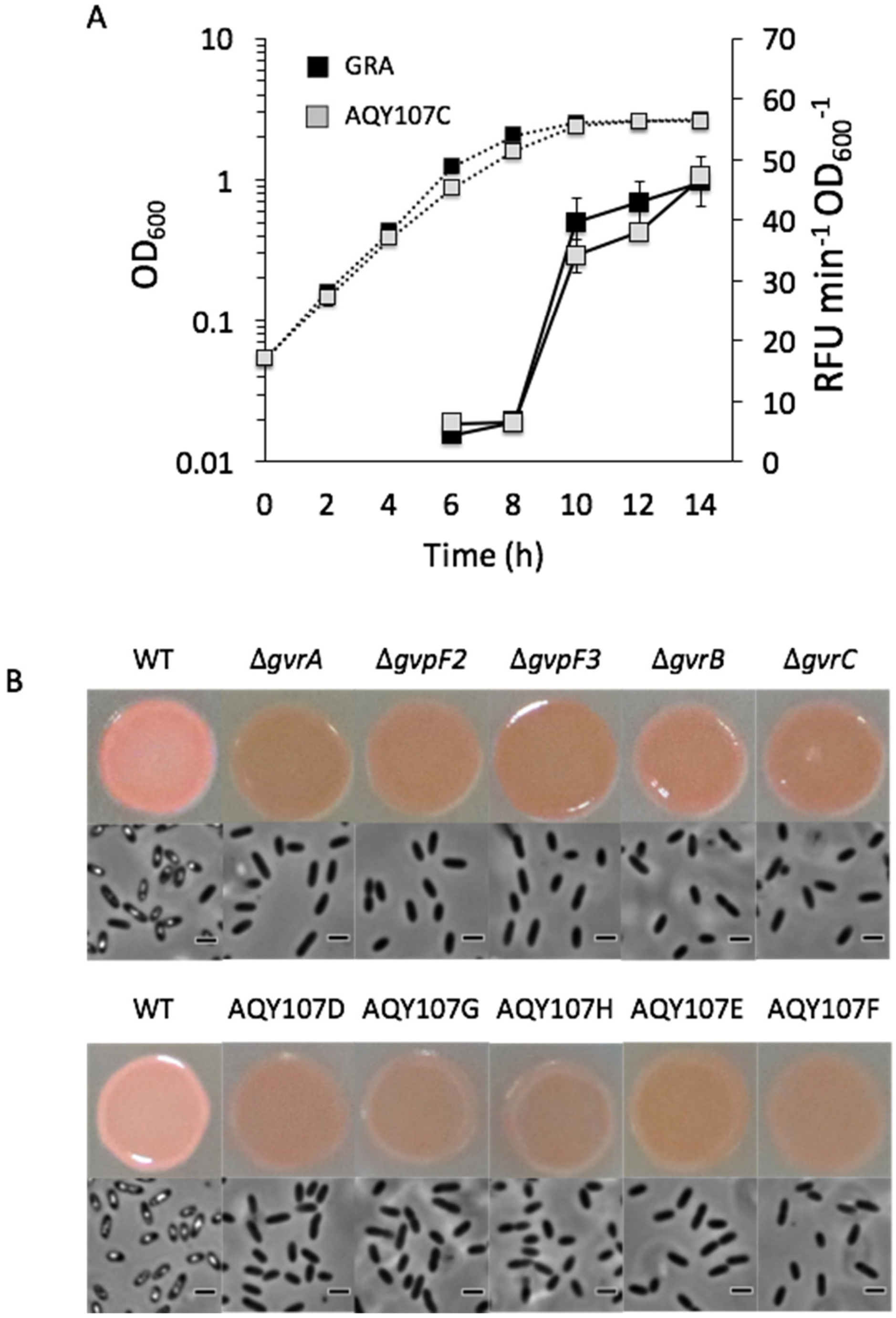

Figure S2. Effect of *trkH* mutation on the *gvrA*-*gvrC* operon. A. *gvrA* transcription activity in GRA (*gvrA*::*uidA*) and AQY107C (*gvrA*::*uidA, trkH::*TnKRCPN1) (Table S1). Growth (dotted lines) was measured as OD_{600} and gene reporter activity (continuous lines) as RFU min^{-1} OD_{600}^{-1}. ANOVA analysis of the β-glucuronidase reporter activity from 6 to 14 h of growth $F = 3.17 > F_{crit} = 4.35$; *p*-value 0.09. These data represent the average value of biological replicates (n=3, error bars show standard deviation). B. Patch morphology and PCM of patches cells with mutations in *trkH* and GV essential genes from the *gvrA*-*gvrC* operon. *trkH* mutant cells AQY107D, G, H, E and F in-frame mutations in gvrA, *gvpF2, gvpF3, gvrB* and gvrB, respectively (Table S1). Scale bars correspond to 1 μm.

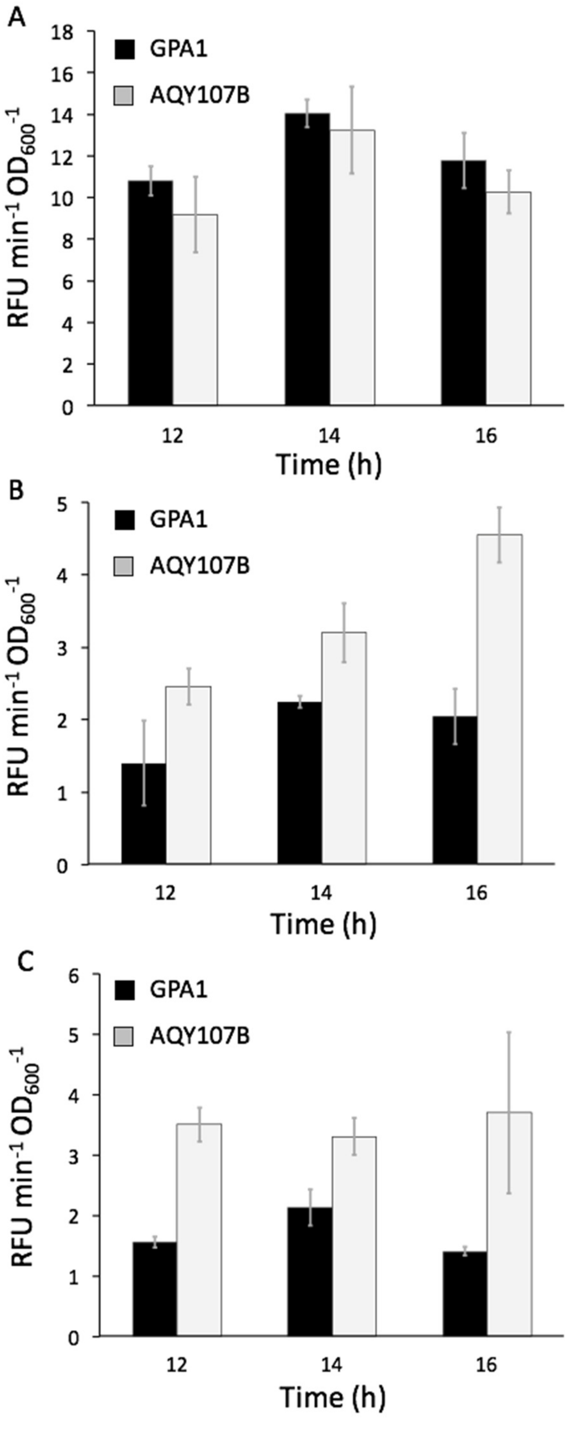

Figure S3. *gvpA1* expression in minimal media with an alternate potassium source to KCl. Reporter fusion strains GPA1 (*gvpa1*::*uidA*) and AQY107B (*gvpa1*::*uidA trkH*::TnKRCPN1) (Table S1) were grown in minimal media at final concentrations of (A) 0.14 mM, (B) 1.4 mM and (C) 14 mM K^+ using minimal medium with potassium buffer instead of KCl as a source of K^+. ANOVA analysis of the β-glucuronidase reporter activity from 12 to 16 h of growth with (A) $F = 4.08 < F_{crit} = 4.74$; p-value 0.066; (B) $F = 70.87 > F_{crit} = 4.74$; p-value $2.22*10^{-6}$, and (C) $F = 42.57 > F_{crit} = 4.74$; p-value $2.83*10^{-5}$. These data represent the average value of biological replicates (n=3, error bars show standard deviation).

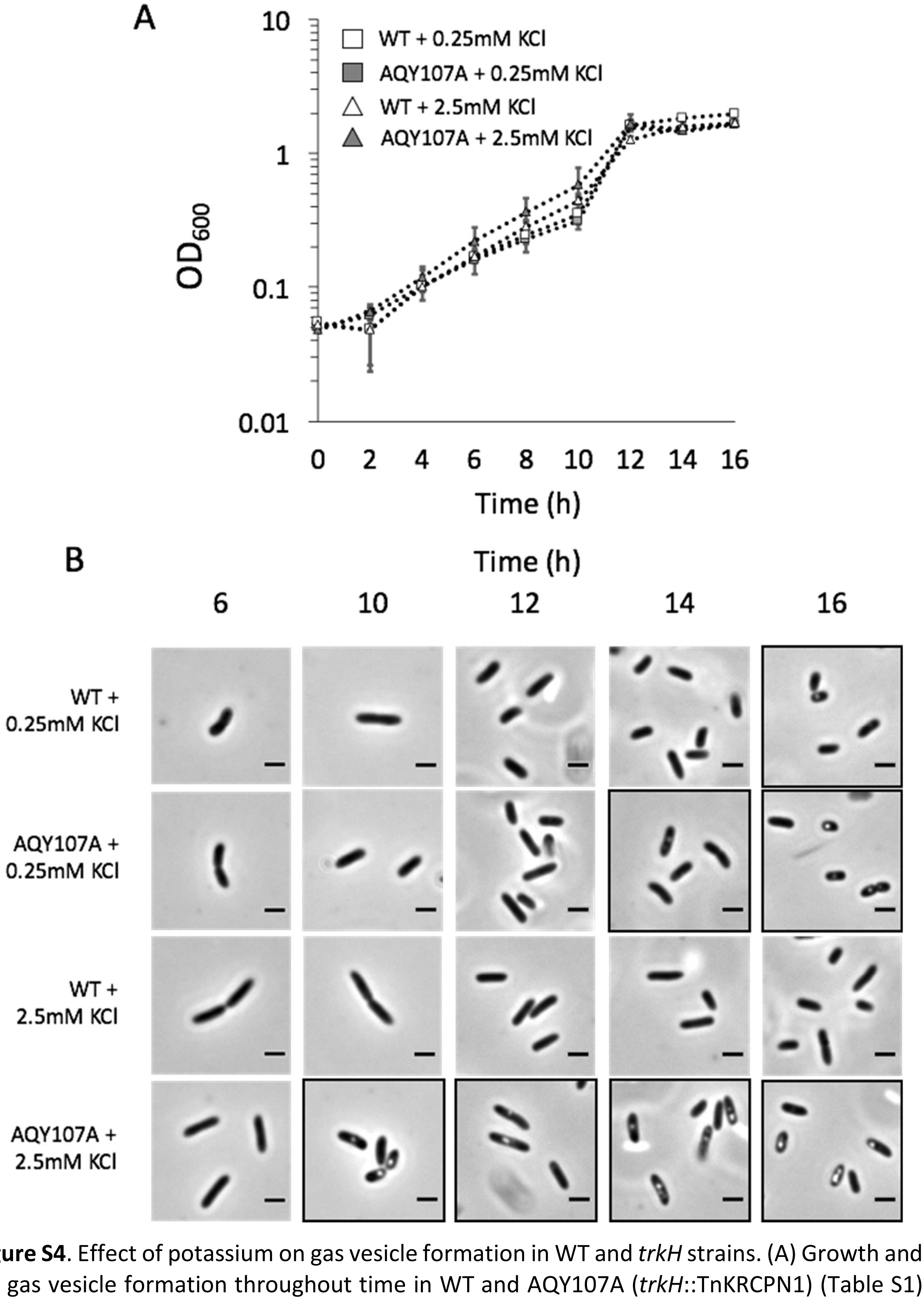

Figure S4. Effect of potassium on gas vesicle formation in WT and *trkH* strains. (A) Growth and (B) gas vesicle formation throughout time in WT and AQY107A (*trkH*::TnKRCPN1) (Table S1) cells grown in the presence of 0.25 mM and 2.5 mM KCl. Images of cells with GVs are framed with black lines. PCM images were taken immediately after OD_{600} measurements. These data represent the average value of biological replicates (n=3, error bars show standard deviation).

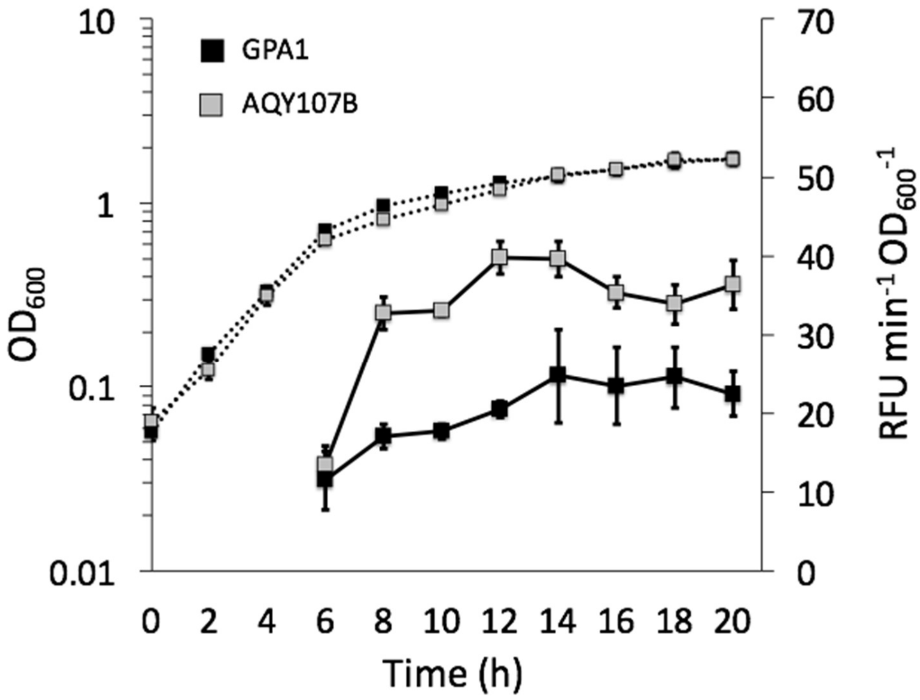

Figure S5. *gvpA1* expression in the *trkH* mutant under microaerophilic conditions. Growth (dotted lines) was measured as OD_{600} and reporter activity (continuous lines) as RFU min^{-1} OD_{600}^{-1} in reporter fusion strains GPA1 (*gvpa1*::*uidA*) and AQY107B (*gvpa1*::*uidA* *trkH*::TnKRCPN1) (Table S1). ANOVA analysis of the β-glucuronidase reporter activity from 6 to 20 h of growth $F = 86.86 > F_{crit} = 4.35$; *p*-value $1.02*10^{-8}$. These data represent the average value of biological replicates (n=3, error bars show standard deviation).

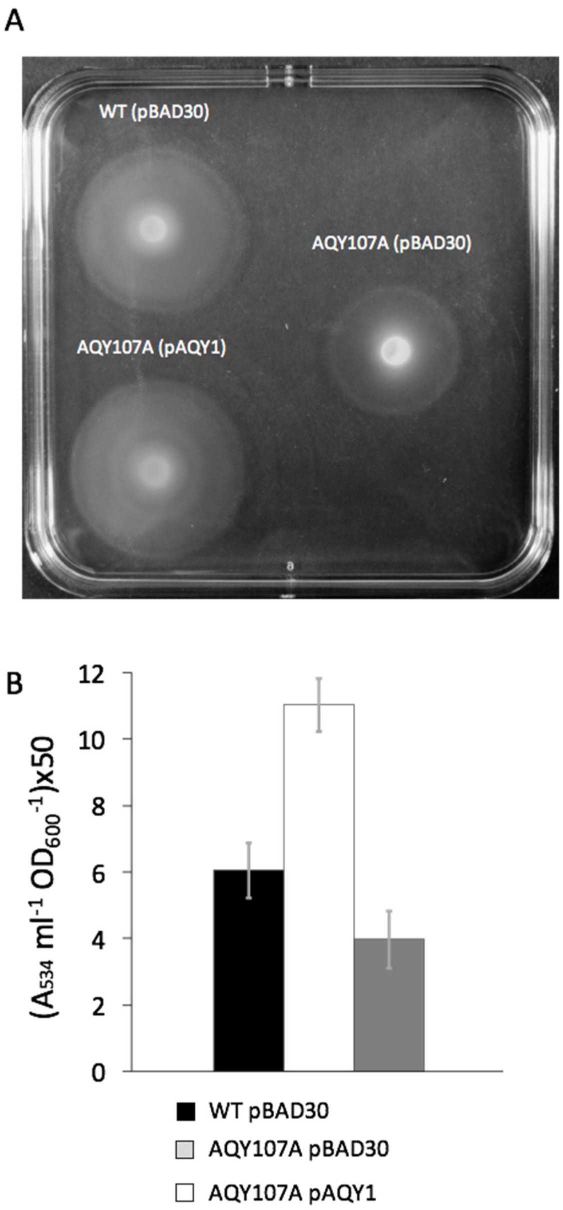

Figure S6. TrkH is a pleiotropic regulator. Complementation of (A) swimming motility and (B) prodigiosin production (A534 ml^{-1} OD_{600}^{-1}) in the *trkH* mutant. WT and AQY107A (*trkH*::TnKRCPN1) carrying pBAD30 (empty vector) (Table S1) were used as controls. AQY107A was complemented with pAQY1 (Table S1). (A) The image is representative of three biological replicates. (B) These data represent the average value of biological replicates (n=3, error bars show standard deviation).

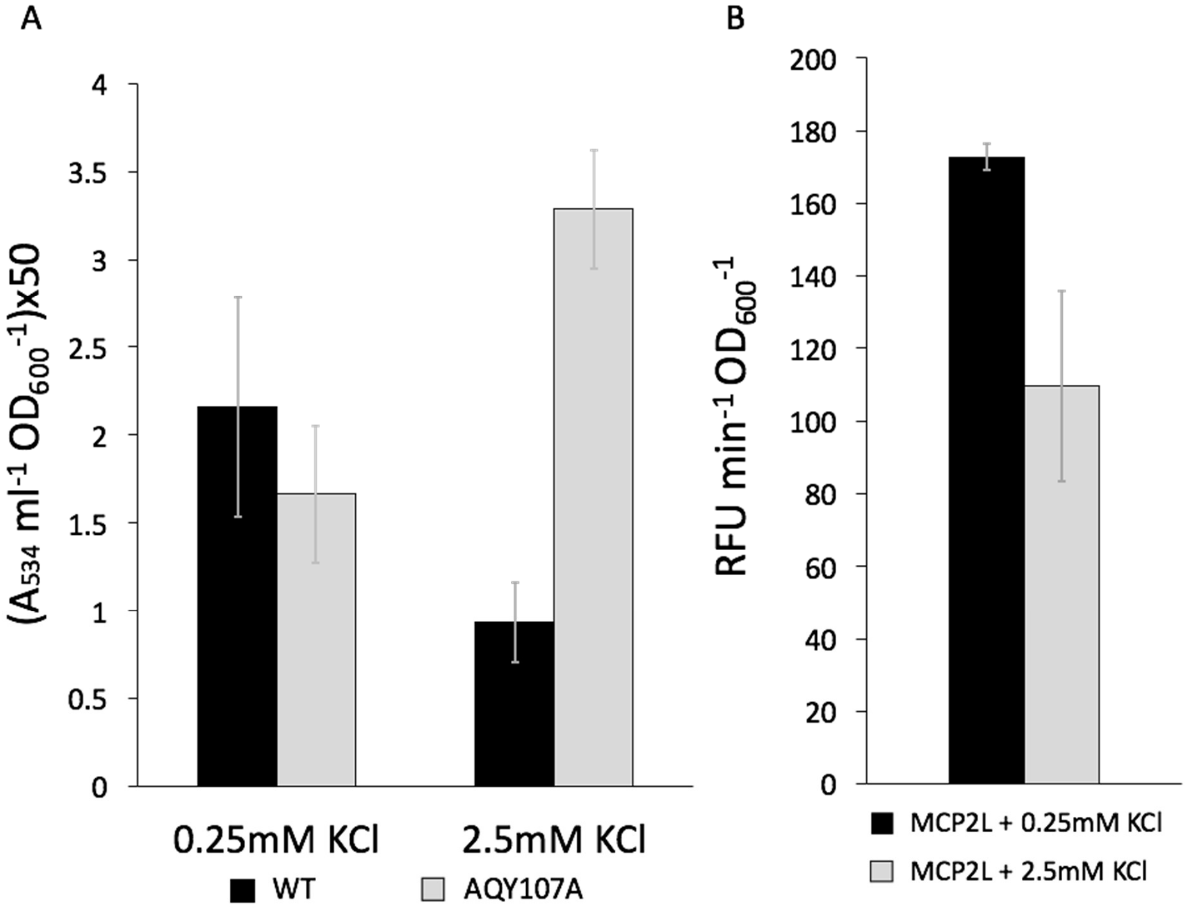

Figure S7. Potassium and TrkH are negative regulators of prodigiosin production. A. Pigment production of WT and AQY107A (*trkH*::TnKRCPN1) (Table S1). B. β-galactosidase reporter activity in fusion strain MC2PL (*pigA*::*lacZ*) (Table S1). WT, AQY107A and MCP2L cells were grown in minimal media with different potassium concentrations and measurements were taken after 12 h. These data represent the average value of biological replicates (n=3, error bars show standard deviation).

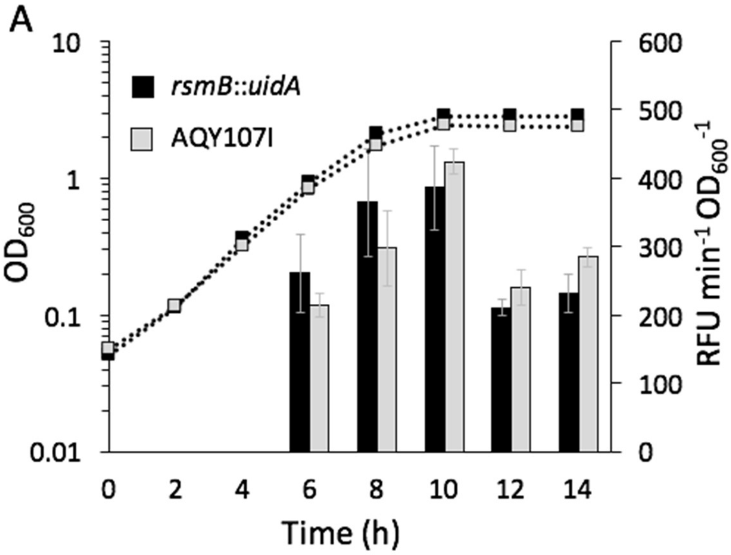

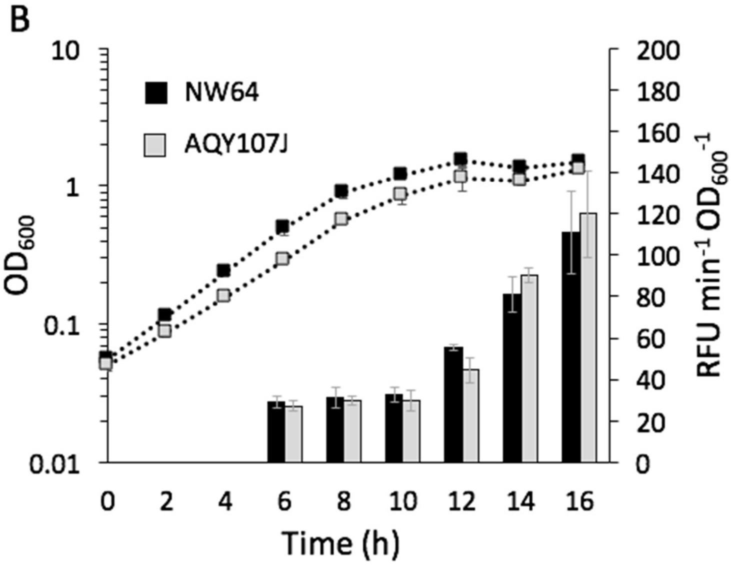

Figure S8. The mutation in *trkH* did not alter *rsmB* and *rsmA* transcription. Growth (dotted lines) and the β-glucuronidase gene reporter acctivity (bars) in (**A**) *rsmB*::*uidA* and AQY107I (*rsmB*::*uidA, trkH*::TnKRCPN1), and (**B**) NWA64 (*rsmA*::*uidA*) and AQY107J (*rsmA*::*uidA, trkH*::TnKRCPN1) (Table S1). Cells were grown in LB media. These data represent the average value of biological replicates (n=3, error bars show standard deviation).

Supplementary references.

1. Thomson, N.R., Crow, M.A., McGowan, S.J., Cox, A., and Salmond G.P. (2000) Biosynthesis of carbapenem antibiotic and prodigiosin pigment in *Serratia* is under quorum sensing control. *Mol Microbiol* 36:539-56.
2. Ramsay, J.P, Williamson, N.R, Spring, D.R., and Salmond, G.P. (2011) A quorum-sensing molecule acts as a morphogen controlling gas vesicle organelle biogenesis and adaptive flotation in an enterobacterium. *Proc Natl Acad Sci USA* 108:14932-7.
3. Tashiro, Y., Monson, R.E., Ramsay, J.P., and Salmond GP. (2016) Molecular genetic and physical analysis of gas vesicles in buoyant enterobacteria. *Environ Microbiol* 18:1264-76.
4. Slater, H., Crow, M., Everson, L., and Salmond, G.P. (2003) Phosphate availability regulates biosynthesis of two antibiotics, prodigiosin and carbapenem, in *Serratia* via both quorum-sensing-dependent and-independent pathways. *Mol Microbiol* 47:303-20.
5. Hampton, H.G., McNeil, M.B., Paterson ,T.J., Ney, B., Williamson, N.R., Easingwood, R.A., et al. (2016) CRISPR-Cas gene-editing reveals RsmA and RsmC act through FlhDC to repress the SdhE flavinylation factor and control motility and prodigiosin production in *Serratia*. *Microbiology* 162:1047.
6. Demarre, G., Guérout, A.M., Matsumoto-Mashimo, C., Rowe-Magnus, D.A., Marlière, P., and Mazel, D. (2005) A new family of mobilizable suicide plasmids based on broad host range R388 plasmid (IncW) and RP4 plasmid (IncPα) conjugative machineries and their cognate *Escherichia coli* host strains. *Res Microbiol* 156:245-55.
7. Evans, T.J., Crow, M.A., Williamson, N.R., Orme, W., Thomson, N.R., Komitopoulou, E., and Salmond, G.P. (2010) Characterization of a broad-host-range flagellum-dependent phage that mediates high-efficiency generalized transduction in, and between, *Serratia* and *Pantoea*. *Microbiology* 156:240-7.
8. Monson, R., Smith, D.S., Matilla, M.A., Roberts, K., Richardson, E., Drew, A., et al. (2015) A plasmid-transposon hybrid mutagenesis system effective in a broad range of Enterobacteria. *Front Microbiol* 6:1442.
9. Guzman, L.M., Belin, D., Carson, M.J., and Beckwith, J.O. (1995) Tight regulation, modulation, and high-level expression by vectors containing the arabinose PBAD promoter*. J Bacteriol* 177:4121-30.
10. Fineran PC, Williamson NR, Lilley KS, and Salmond GP. (2007) Virulence and prodigiosin antibiotic biosynthesis in *Serratia* are regulated pleiotropically by the GGDEF/EAL domain protein, PigX. *J Bacteriol* 189:7653-62.
